# Thada Is Dispensable for Female Fertility in Mice

**DOI:** 10.3389/fendo.2022.787733

**Published:** 2022-04-05

**Authors:** Shan Han, Yuqing Zhang, Yukun Zheng, Congcong Liu, Yonghui Jiang, Shigang Zhao, Han Zhao

**Affiliations:** ^1^ Center for Reproductive Medicine, Cheeloo College of Medicine, Shandong University, Jinan, China; ^2^ Key laboratory of Reproductive Endocrinology of Ministry of Education, Shandong University, Jinan, China

**Keywords:** polycystic ovarian syndrome, ovarian function, follicle development, *in vivo*, THADA

## Abstract

Genome-wide association studies (GWAS) have identified *THADA* as one of the susceptibility genes for polycystic ovary syndrome (PCOS). Single nucleotide polymorphisms (SNPs) in the *THADA* gene showed significant over-transmission in PCOS and strong correlations with testosterone level. However, there was insufficient evidence to verify the effect of *THADA in vivo* on female reproductive system. In this study, we investigated the impacts of *Thada* ablation on ovarian function and reproductive outcomes with knockout (KO) mice. The results showed that the *Thada* deletion was insufficient to affect ovarian folliculogenesis, steroidogenesis, and female fertility. Additionally, we stressed the mice with high-fat-high-sugar diet (HFHS). In this case, the KO mice still merely had a negligible impact on ovarian function. These findings indicated that *Thada* deficiency was dispensable for female fertility in mice, which enriched our knowledge about *in vivo* functions of PCOS susceptibility genes.

## Introduction

Polycystic ovary syndrome, the most common endocrine disorder in reproductive aged women, is characterized by hyperandrogenism, oligomenorrhoea, amenorrhea, and polycystic ovaries (PCOM), combined with multiple metabolic abnormalities (1). PCOS is currently considered as a multifactorial disease affected by environmental and polygenic factors (2). Although environmental triggers have been generalized researched, specific environmental exposures still hardly explain the widespread global prevalence and the familial inheritance of PCOS (3). For this reason, the important role of genetic factors has received considerable attention, despite the difficulty of research process. Benefiting from the sophistication of GWAS technology, multiple PCOS genetic susceptibility loci have been identified. Our group firstly reported the 11 PCOS association loci in Han Chinese, many of which were replicated in European and American population (4, 5). Of the loci consistently present in the different GWAS reports, the one with the strongest association mapped to the genomic region of *THADA*. In addition, a series of recent researches have demonstrated *THADA* carried risk alleles associated with reproductive endocrine disturbances in PCOS patients. Using a recessive model, Cui et al. found that the SNPs in *THADA* were associated with increased testosterone (T) levels in subjects with PCOS (6). Besides that, SNP rs13429458, in the *THADA* gene, showed over transmission in Han Chinese PCOS cases (7). It is proposed that *THADA* might participate in the occurrence and development of PCOS.

*THADA* encodes thyroid adenoma associated protein, which is expressed broadly in various tissues such as thyroid, brain and ovary (8). *THADA* has been identified as a candidate gene in PCOS for many years and the vast majority of researches calculated the correlation but failed to prove causation. The THADA’s normal physiological functions still remained enigmatous, especially in female reproduction process. There was only one report found that *THADA* knockout *Drosophila* were obese and produced less energy (9). To gain insight into the physiological functions of THADA *in vivo*, we constructed *Thada* knockout mice and focused on its effects on reproductive system.

## Materials and Methods

### Animals

We selected exon 8 to exon 11 as target sites to generate the *Thada* founder mice by the CRISPR-Cas9 technology. We used the heterozygous mutants mice (*Thada^+/−^*) intercrossed, and successfully obtained homozygous mice (*Thada^-/-^*). Littlermate wild type mice (WT) were included as controls. Genotyping of *Thada* knockout mice was done by PCR on tail genomic DNA.

All mice described were maintained on the C57BL/6J background. All animals were fed in an ad libitum mode, using a commercial pelleted diet (Beijing Keao Xieli Feed Co.). Mice were housed in groups (5 adult mice per cage) under specific pathogen-free conditions in a temperature controlled room (21-22°C) with a 12 h-12 h light/dark cycle. Beginning at Pnd 21, vaginal opening was monitored daily. Mice used for HFHS experiments were fed with normal chew diet until 8 weeks of age, after which they were maintained on HFHS diet (Research Diet 12451) for another 10 weeks. Female mice were sacrificed between 3 and 6 months.

### Estrous Cycle and Fertility Assay

At 8 weeks of age, the estrous cycle of WT and KO mice was identified by vaginal smear once a day for 2 weeks. The percentage of different stages (diestrus, proestrus, estrous, and metestrus) of the estrous cycle was determined and recorded. The fertility measurement of mice started at the age of 8 weeks and lasted for six months. WT and *Thada* KO female mice were mated with fertile male mice of the same age using a ratio of 2:1 and checked daily for signs of pregnancy. The number of pups per litter and the gender ratio were calculated.

### Blood Measurements

Serum samples were collected from mice in basal resting condition during diestrus. After coagulation, the serum samples were extracted after centrifugation 2500rpm for 10min (4°C) and stored at −80°C until used. Serum testosterone, progesterone, and estradiol were measured with radioimmunoassay (Beijing North Institute of Biological Technology).

### Ovarian Gonadotropin Stimulation

*Thada* knockout and wild type mice were first injected intraperitoneally with 7.5 IU of pregnant mare serum gonadotropin (PMSG) and after 44 h with 7.5 IU of human chorionic gonadotropin (hCG) (Ningbo Sansheng Pharmaceutical Co., Ltd., China). Sixteen hours later, animals were sacrificed by decapitation. The cumulus-oocyte-complexes were extracted from the fallopian tubes and the oocytes were counted after hyaluronidase treatment. The mature oocytes were assessed by the extrusion of the first polar body (PB1).

### Ovarian Histology and Follicle Counting

Ovaries were fixed overnight with 4% PFA, then dehydrated and embedded in paraffin. For hematoxylin and eosin (H&E) staining, the sections were stained according to standard histological protocols. The images were captured by an inverted microscope.

For follicle counting, the ovaries of normal diet mice were serially sectioned (5μm) and stained with H&E. Every fifth section of each ovary was observed under a microscope. Follicles were classified as healthy or atretic by morphological criteria described previously (10). Atretic preantral follicles consisted of the oocyte that had degenerated with either a disorganized granulosa layer with apoptotic cells (more than 5% of the cells showed apoptosis signs) and/or a hypertrophied theca layer surrounded. Atretic antral follicles were usually present as an intact oocyte, whereas the layer of granulosa cells contained more than 5% apoptotic cells, and the theca layer showed signs of hypertrophy. All kinds of atretic follicles were excluded during the count. Follicles were classified as described previously (11). Only primordial, primary, secondary, and antral follicles were counted when the oocyte with nucleus and nucleolus were present in every fifth section. The adjacent sections were examined, and the follicle with the same nucleus was counted only once during the counting of larger follicles. The number of primordial, primary, secondary, and antral follicles was multiplied by five to estimate the total number of follicles within one ovary. Every tenth section of each ovary was observed to determine the number of corpus luteum. In order to prevent double-counting of corpus luteum, adjacent sections were traced and compared.

### RNA Extraction and RT-qPCR Analysis

Genotyping of *Thada* knockout mice was done by PCR on tail genomic DNA. The primers for PCR genotyping are:

*Thada*-allele forward primer: 5’-CAGATGGGATACAGAGGCTAGGCG-3’; reverse primer: 5’-AATGACTGGAGATAATTGCTCATGCC-3’;

WT-allele forward primer: 5’-CAGATGGGATACAGAGGCTAGGCG-3’; reverse primer: 5’-CGCACTCTTTCCCACCTGTCTTC-3’.

Ovarian tissues were collected, snap-frozen in liquid nitrogen, and stored at -80°C. Total RNA was extracted with Trizol, 1μg of RNA were reverse transcribed using the PrimeScript RT Reagent Kit (Takara, Shiga, Japan). Real-time PCR was performed using the LightCycler 480 Probes Master kit (Roche Diagnostics, Meylan, France).

### Statistical Analysis

The results are presented as the mean ± SEM; each experiment was repeated at least 3 times. The significant differences between groups were calculated by two-tailed unpaired Student’s t-test and Chi-square test. *P*-value<0.05 was considered significant.

## Result

### *Thada* Knockout Mice Have Normal Fertility

**Figure 1:**
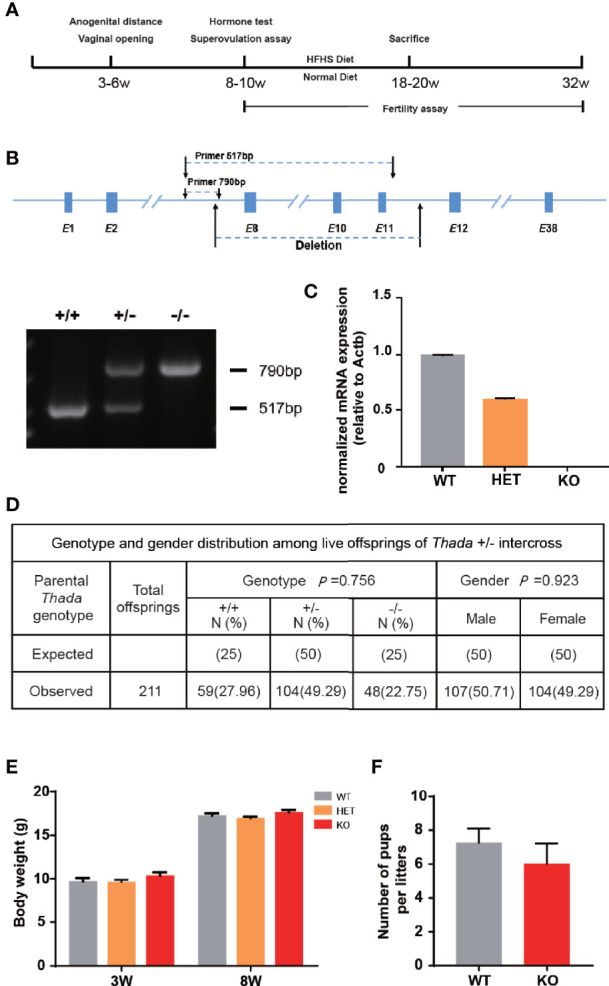
*Thada* knockout mice have normal fertility. **(A)** The flowchart of experimental design. **(B, C)**. Generation and dentification of *Thada* knockout mice. Efficiency of knockdown in ovary was determined by qPCR. **(D)** The genotype distribution and the male/female ratio of offsprings were obtained by mating *Thada* ^+/-^ (n = 211). **(E)**. KO and WT had similar body weights (n = 15-24). **(F)** WT and KO female mice had the similar litter size (n = 5).

To explore the functional role of *Thada in vivo*, we constructed a *Thada* global knockout (*Thada^-/-^*) mouse model and designed the experiments (**Figures 1A, B**). *Thada* was undetectable in ovarian tissues of KO mice at the RNA level, and heterozygotes also resulted in a 50% reduction (**Figure 1C**). The genotype distribution of offsprings obtained by mating *Thada^+/-^* conformed to Mendelian inheritance and the male/female ratio was 1.03 (**Figure 1D**). This reduction of *THADA* levels did not affect intrauterine development or sex selection. In addition to this, the development of KO mice appeared normal compared with WT mice from birth to adulthood. There were no significant differences in the body weight at 3 weeks and 8 weeks between WT and KO mice (**Figure 1E**). The fertility of KO and WT female mice was evaluated. The litter size and the number of males/females per litter were comparable between the two genotypes (**Figure 1F**). These results suggested that *Thada* with altered expression did not result in growth retardation or adverse pregnancy outcomes.

### No Detectable Ovarian Dysfunction in KO Mice Fed With Normal Diet

**Figure 2:**
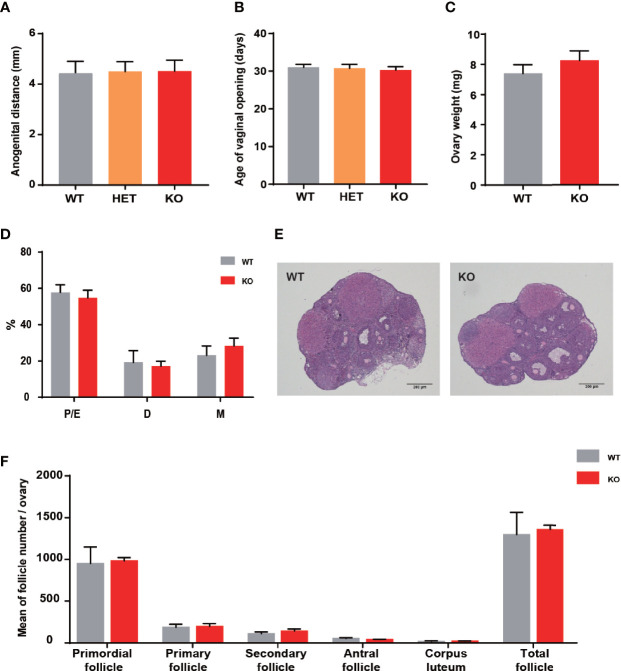
*Thada* knockout mice did not present ovarian development abnormalities. **(A)** Anogenital distance (AGD) of 4 week-old mice (n = 4-7). **(B)** Vaginal opening time was similar between WT and KO mice (n = 4-7). **(C)** The ovarian weights were no different between WT and KO mice (n=8-10). **(D)** Estrous cycle profile of WT and KO mice (Proestrus-Estrus P/E), Matestrus M, Diestrus D) (n = 6-9). **(E)** Hematoxylin-Eosin stained ovaries sections of WT and KO mice, scale bar=200μm. **(F)** The numers of these follicles at different developmental stages of maturation(n = 3-4).

There was a closed association between *THADA* and hyperandrogenism in previous studies. Therefore, the development and function of the ovary have become essential aspects of our research. The anogenital distance was the evaluation index of hyperandrogenism, and the distances were similar between KO and WT mice (**Figure 2A**). The vaginal opening was considered a reliable marker for puberty onset. It did not exhibit differences between KO and WT mice (**Figure 2B**), and the time of appearance of the first estrus was also temporally coincided in KO and WT mice. In addition, the ovarian weights of the two groups were approximately the same (**Figure 2C**). These results illustrated that KO mice experienced normal pubertal development through early life.

**Figure 3:**
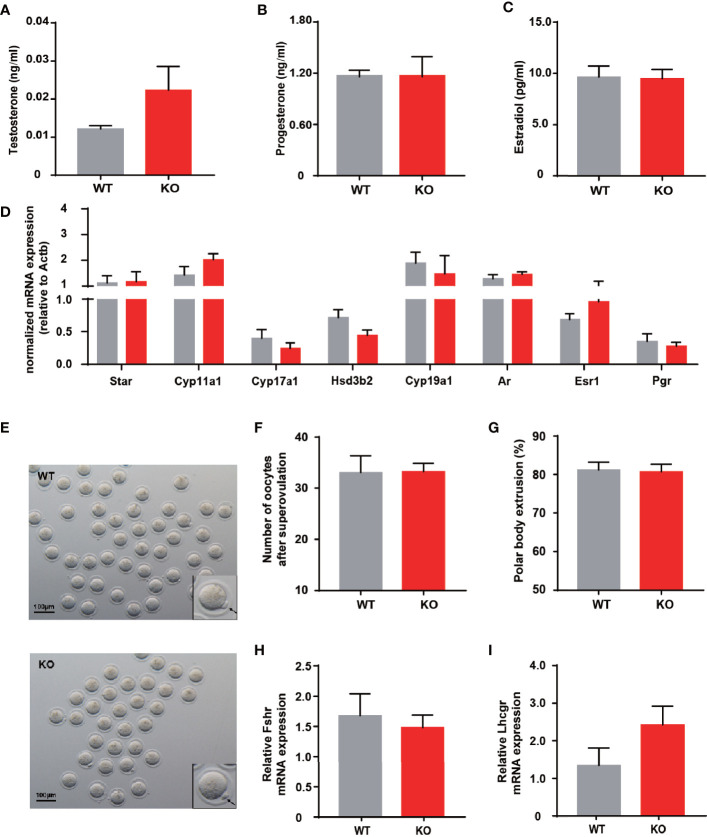
*Thada* knockout mice have normal ovarian function and ovulation. **(A–C)** Serum levels of testosterone, progesterone and estradiol of adult female WT and KO mice (n = 9-10). **(D)** qRT-PCR analysis of hormone related genes in ovary (n = 8-9). **(E)** Morphology ofMII oocytes, where black arrows indicate polar bodies, scale bar=100μm.(n = 4-6) **(F)** Number of oocytes retrieved after ovarian stimulation. **(G)** Polar body extrusion rates in oocytes from WT and KO mice at adult stage (n = 4-6). **(H, I)**. mRNA expression of gonadotrophin receptors in ovary(n = 8).

Impaired ovulation is another common reason for patients to access the clinic. Estrous cycles, follicles development, hormone levels, and ovarian response to gonadotropin stimulation can reflect the function of the ovaries. Compared to WT mice, KO mice showed comparable estrous cyclicity and cycle length, with all phases of the estrous cycle present (**Figure 2D**). In keeping with WT mice, the ovaries of KO mice exhibited appropriate proportions of follicles at different stages (**Figures 2E, F**). Despite circulating testosterone levels were mildly elevated in adult KO mice, there was no statistical difference between the two genotypes (**Figure 3A**). Serum estrogen and progesterone levels of KO mice achieved comparable levels of WT mice, consistent with the results observed in the ovaries (**Figures 3B, C**). At the same time, we quantified the expression of hormone-related genes and detected no meaningful differences (**Figure 3D**). After being stimulated by PMSG and hCG, KO mice did not demonstrate alteration in the number of oocytes retrieved or PB1 extrusion rate (**Figures 3E, H**). The mRNA expression levels of gonadotropin receptors in the ovaries also corroborated the results of the superovulation experiment, with levels of Fshr and Lhcgr not different between the two groups (**Figures 3H, I**). These results suggested that the ovaries of *Thada* knockout mice may have normal function. Indeed, despite the *THADA* SNP rs13429458 being involved in PCOS, investigators did not find significant differences in reproductive indicators, such as serum LH level or FSH level, between genotypes of PCOS patients (12). It demonstrated that the reproductive pathogenicity of a single gene alteration was relatively limited.

### HFHS Could Not Induce Reproductive Dysfunctions in KO Mice

**Figure 4:**
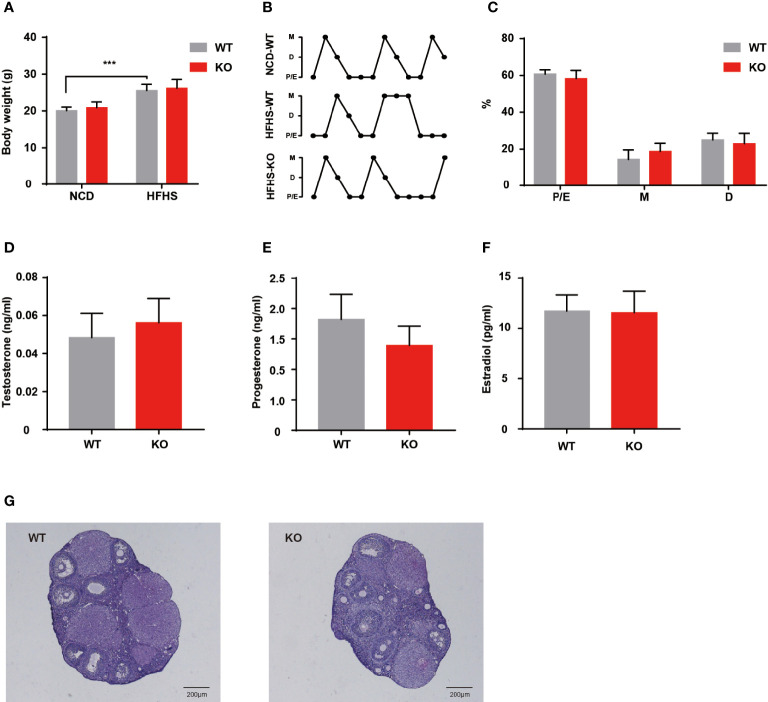
HFHS could not induce reproductive dysfunctions in KO mice. **(A)** Body weights were measured for WT and KO mice with normal chow diet or HFHS diet (n = 6-10). **(B, C)** Estrous cycle profile of WT and KO mice with ten weeks HFHS-fed. WT mice with the normal chow diet were selected as control groups (n = 7-8). **(D–F)**. Serum levels of testosterone, progesterone, and estradiol of HFHS-fed mice (n = 7-8). **(G)** HE stained histological sections of ovaries, scale bar=200μM. ***P < 0.005.

Environmental factors, especially dietary factors, strongly influence PCOS patients. Animal studies also have shown that HFHS diet altered ovarian morphology and even induces the formation of polycystic ovaries (13). We performed HFHS diet induction to observe the reproductive function of Thada knockout mice. After ten weeks of HFHS treatment, the body weight increased in both genotypes, but there was no difference in change between WT and KO groups (**Figure 4A**). A subset of indicators for evaluating ovarian function was examined, such as serum hormone levels, estrous cyclicity, and ovarian morphology. Despite the period length of each estrous cycle prolonged by HFHS-fed, KO mice still exhibited similar fluctuations compared to the WT (**Figures 4B, C**). There were no notable differences in hormone levels between the two genotypes in the basal state (**Figures 4D–F**). Meanwhile, there was little change in ovarian morphology and structure corresponding to hormones (**Figure 4G**). These results suggested that *Thada* knockout mice maintain relatively stable ovarian function under metabolic stress.

## Discussion

*THADA* was a promising candidate gene for PCOS in our previous work, but researches on the *in vivo* function of *Thada* in mammals were limited reported. Therefore, the *Thada* knockout mouse model provided an opportunity to explore its function in the female reproductive system. The effect of *Thada* on fertility and ovarian function was the focus of this study. Prior GWAS evidence suggested that the rate of abortion and the number of oocytes retrieved of patients with the *THADA* rs12478601 CC genotype increased, but the rate of clinical gestation decreased (14, 15). In genotype-phenotype correlational analysis, the *THADA* gene contributed to hyperandrogenism in PCOS. However, *Thada* knockout mice failed to replicate these results obtained in humans. *Thada* deletion did not lead to abnormal growth and development. The survival rate and bodyweight of KO female mice were at par with WT mice. *Thada* KO mice could complete puberty at a usual time and had relatively normal fertility in adulthood. Only circulating testosterone level was detected mildly elevated in adult KO mice. The inability of mouse models to explain the correlations found in humans suggested that several vital considerations need addressing when studying the function of risk genes for PCOS.

PCOS is a complex and heterogeneous reproductive endocrine disorder. Therefore, it was not easy to assess the impact of genetic factors on regulating reproductive functions. Earlier studies have established a genetic predisposition in PCOS and several genes involved in ovarian function (1, 16), but none was strong enough to correlate alone with susceptibility to the disease (17, 18). Our results also supported the perspective that the combination of polygenic, epigenetic, and developmental factors determines a person’s susceptibility to PCOS (2). In addition to the limited pathogenicity of genetic factors, *THADA* may affect different tissues and organs, or even opposed effects, because of its broad expression (19). In evolutionary-related studies, the pathogenic mechanisms underlying PCOS might be candidate factors for survival advantage of the human being (17, 18). *THADA* may have played a positive role in racial reproduction and adaptability to the environment. Previous reports showed that *THADA* also affects pancreatic function and thermogenesis (9, 20). It is reasonable to speculate that *Thada* seemed to play a more crucial role in regulating metabolic homeostasis rather than the reproductive system.

Limitations of the study should also be considered. Caution is required in extrapolating experimental data from one species to another, especially for a highly heterogeneous disease like PCOS (21). The mouse was a type of multiovulatory animal, and its follicle development was significantly different from that of humans (22). In various animal models of PCOS, rhesus or sheep tended to exhibit more pronounced reproductive phenotypes than mice (23–25). However, due to technical and other limitations, the mouse remained the preferred animal for studying single-gene function. Another limitation is that while HFHS can impair ovarian function and even exacerbate PCOS progression, HFHS may not be an environmental factor that interacts with THADA or does not directly affect the progeny itself. In a current study, *THADA* knockout *Drosophila* were obese, hyperphagic, had reduced energy production, and were sensitive to the cold (9). Therefore, cold stimulation or prenatal exposure to HFHS will be the next step in our research.

Our work further validated the concept that the combination of genetic and nongenetic factors defined a person’s susceptibility to developing PCOS rather than a single genetic alteration. Future studies regarding PCOS susceptibility genes should consider the effects of dietary, inflammatory, stress, and other factors. Thus, many aspects of *THADA* remained worthy of further investigation, although the knockout mice currently do not show phenotypes in the reproductive system.

## Data Availability Statement

The raw data supporting the conclusions of this article will be made available by the authors, without undue reservation.

## Ethics Statement

The animal study was reviewed and approved by the Animal Ethics Committee of the School of Medicine, Shandong University.

## Author Contributions

HZ conceived the project and supervised research. SH and YZha designed the experiments and wrote the manuscript. SH, YZhe, CL, and YJ performed the experiments and analyzed data. SZ constructed the knock mice. All authors contributed to the article and approved the submitted version.

## Funding

This study was supported by the National Key Research and Development Program of China (2021YFC2700400, 2021YFC2700701), Basic Science Center Program of NSFC (31988101), National Natural Science Foundation of China(82100839), and China Postdoctoral Science Foundation (2018M640639, 2020T130373).

## Conflict of Interest

The authors declare that the research was conducted in the absence of any commercial or financial relationships that could be construed as a potential conflict of interest.

## Publisher’s Note

All claims expressed in this article are solely those of the authors and do not necessarily represent those of their affiliated organizations, or those of the publisher, the editors and the reviewers. Any product that may be evaluated in this article, or claim that may be made by its manufacturer, is not guaranteed or endorsed by the publisher.
